# Cerebral and pulmonary aspergillosis, treatment and diagnostic challenges of mixed breakthrough invasive fungal infections: case report study

**DOI:** 10.1186/s12879-020-05162-9

**Published:** 2020-07-23

**Authors:** Ali Amanati, Mehrzad Lotfi, Mohammad Sadegh Masoudi, Hadis Jafarian, Fatemeh Ghasemi, Haleh Bozorgi, Parisa Badiee

**Affiliations:** 1 Professor Alborzi Clinical Microbiology Research Center, Shiraz University of Medical Sciences, Namazi Hospital, Zand Ave, Shiraz, 7193711351 Iran; 2 Medical Imaging Research Center, Department of Radiology, Shiraz University of Medical Sciences, Shiraz, Iran; 3 Department of Neurosurgery, Shiraz University of Medical Sciences, Shiraz, Iran; 4 Hematology Research Center, Shiraz University of Medical Sciences, Shiraz, Iran

**Keywords:** *Aspergillus niger*, Brain abscess, *Aspergillus fumigatus*, Breakthrough invasive fungal infections

## Abstract

**Background:**

Breakthrough invasive fungal infections (bIFIs) are an area of concern in the scarcity of new antifungals. The mixed form of bIFIs is a rare phenomenon but could be potentially a troublesome challenge when caused by azole-resistant strains or non-*Aspergillus fumigatus*. To raise awareness and emphasize diagnostic challenges, we present a case of mixed bIFIs in a child with acute lymphoblastic leukemia.

**Case presentation:**

A newly diagnosed 18-month-old boy with acute lymphoblastic leukemia was complicated with prolonged severe neutropenia after induction chemotherapy. He experienced repeated episodes of fever due to extended-spectrum beta-lactamase-producing *Escherichia coli* bloodstream infection and pulmonary invasive fungal infection with *Aspergillus fumigatus* (early-type bIFIs) while receiving antifungal prophylaxis. Shortly after pulmonary involvement, his condition aggravated by abnormal focal movement, loss of consciousness and seizure. Cerebral aspergillosis with *Aspergillus niger* diagnosed after brain tissue biopsy. The patient finally died despite 108-day antifungal therapy.

**Conclusions:**

Mixed bIFIs is a rare condition with high morbidity and mortality in the patients receiving immunosuppressants for hematological malignancies. This case highlights the clinical importance of *Aspergillus* identification at the species level in invasive fungal infections with multiple site involvement in the patients on antifungal prophylaxis.

## Background

Cerebral aspergillosis usually occurs secondary to fungemia after inhaling the fungal spores, proliferating and invading the pulmonary alveolar arteries, after the direct invasion from adjacent structures (sinuses), iatrogenic/penetrating trauma, medical surgery, and contamination of indwelling catheters (ventriculoperitoneal shunts). Multiple brain abscesses could be developed by different mechanisms such as direct angioinvasion, thrombosis, and infarction, mycotic aneurysm, or even intracranial hemorrhage [1–6]. The incidence of cerebral aspergillosis is not known and is directly related to the underlying diseases and host factors. Although the overall prevalence is estimated to be less than 7%, in the high-risk population such as cancer patients the reported frequency is as high as 20–40% [6, 7]. In recent years, the reports of such infections have increased due to the rising number of immunocompromised patients (human immunodeficiency virus infections, patients with chemotherapy or transplant recipients), and improved diagnostic modalities (radiological and microbiological) [3, 8–10].

Breakthrough invasive fungal infection (bIFI) defined as a new fungal infection in the patients receiving therapeutic or prophylactic antifungal agents. Concomitant fungal pneumonia with central nervous system (CNS) infection is a rare occurrence in patients with hematological malignancies, especially those on anti-fungal (AF) prophylaxis. In this report, a mixed pulmonary and CNS aspergillosis reported in a pediatric patient with acute lymphoblastic leukemia (ALL) and we discuss bIFIs during antifungal (AF) prophylaxis.

## Case presentation

**Fig. 1:**
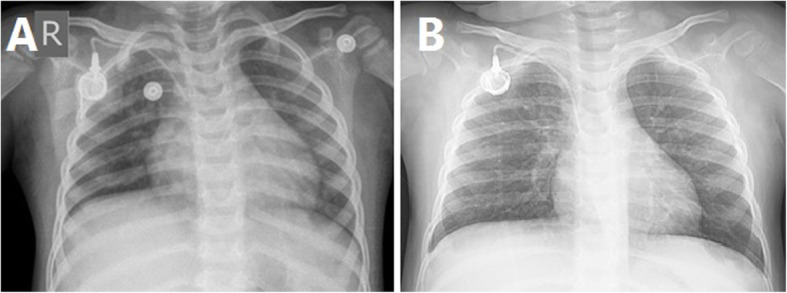
Chest radiograph on day 3 (**a**) and 30 (**b**) after admission without abnormal parenchymal involvement

An 18-month-old boy referred to Amir Medical Oncology Center, Shiraz University of Medical Sciences, Iran, because of scattered bruising patches on his trunk and limbs. He was pale and suffering from mild upper respiratory tract symptoms for several days before admission. In the primary laboratory investigation, severe anemia and thrombocytopenia [Hemoglobin: 4 g/dl (1–6 years: 11.5–13.5 g/dL), platelet count: 10000/mcL (150–400 /microliter) and, white blood cell count (WBC): 106000 cells/mcL (1–6 years: 5000–17,000 cells/microliter)] were detected. B cell ALL diagnosed following bone marrow aspiration/biopsy. Induction chemotherapy started with vincristine, PEG-asparaginase, and daunorubicin. Prophylactic trimethoprim/sulfamethoxazole was initiated simultaneously with his chemotherapy. About 9 days later, his absolute neutrophilic count dropped rapidly (total WBC count: 500 cells/mcL) when he was afebrile. Acute phase reactants were within normal limits (C-reactive protein concentration and erythrocyte sedimentation rate levels were negative and 3, respectively). Prophylactic liposomal amphotericin B (AmBisome, USA) started, and the galactomannan test (GM, *Aspergillus* antigen) was requested (twice per week) for early detection of invasive aspergillosis (IA). Fever developed on the12^th^ day post-admission. Complete sepsis workup was done and meropenem was initiated. Plain X-ray revealed no new parenchymal involvement. Peripheral and central catheter blood cultures performed for the patient using an automated blood culture system (BACTEC medium Becton-Dickinson, Sparks, MD, USA). Both cultures were positive for extended-spectrum beta-lactamase-producing *Escherichia coli* with similar antibiotic susceptibility patterns (sensitive to meropenem, gentamicin, ciprofloxacin, and amikacin). Lock-therapy for catheter bloodstream infection with ciprofloxacin performed (based on hospital protocol). Control blood cultures became negative after 5 days of targeted antibiotic treatment. The chest X-ray was normal at that time (Fig. 1).

**Fig. 2:**
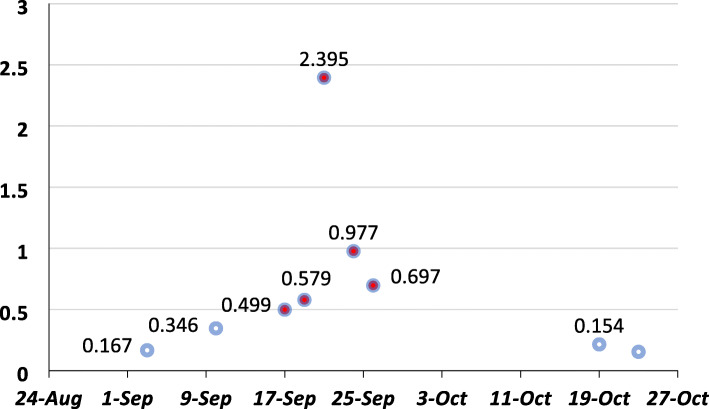
The results of *Aspergillus* serum galactomannan antigen test in the course of admission

**Fig. 3:**
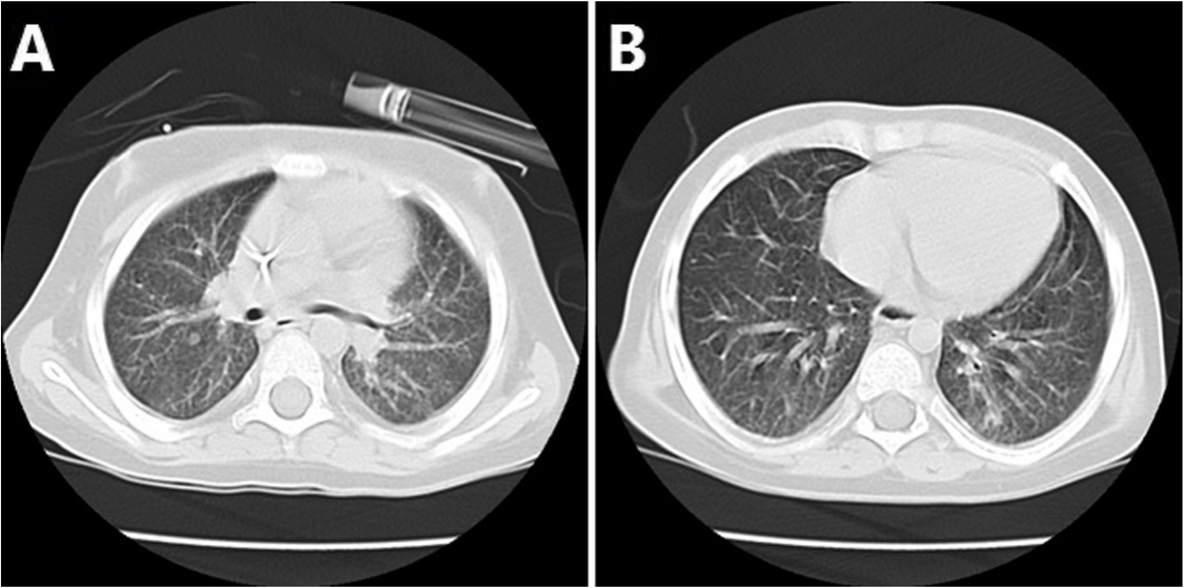
A round nodule about 5 × 5 mm in the superior segment of the right lower lobe (**a**) and another 4 mm nodule in the posterior aspect of the left lower lobe (**b**)

The second episode of fever occurred a few days later despite the complete course of broad-spectrum antibiotic therapy. All cultures repeated, and diagnostic workup upgraded. The stool examined for *Clostridium (C) difficile* toxin gene by polymerase chain reaction (PCR) after culture for *C. difficile*. Quantitative PCR for *Cytomegalovirus* and Mannan test requested in addition to routine indirect mycological (Real-time PCR for *Aspergillus* and *Candida* species) and GM tests. The serum GM test was positive during the second investigation (Fig. 2). Spiral chest computed tomography (CT) scan revealed small ground-glass opacities in the right and left hemithorax (Fig. 3).

**Fig. 4:**
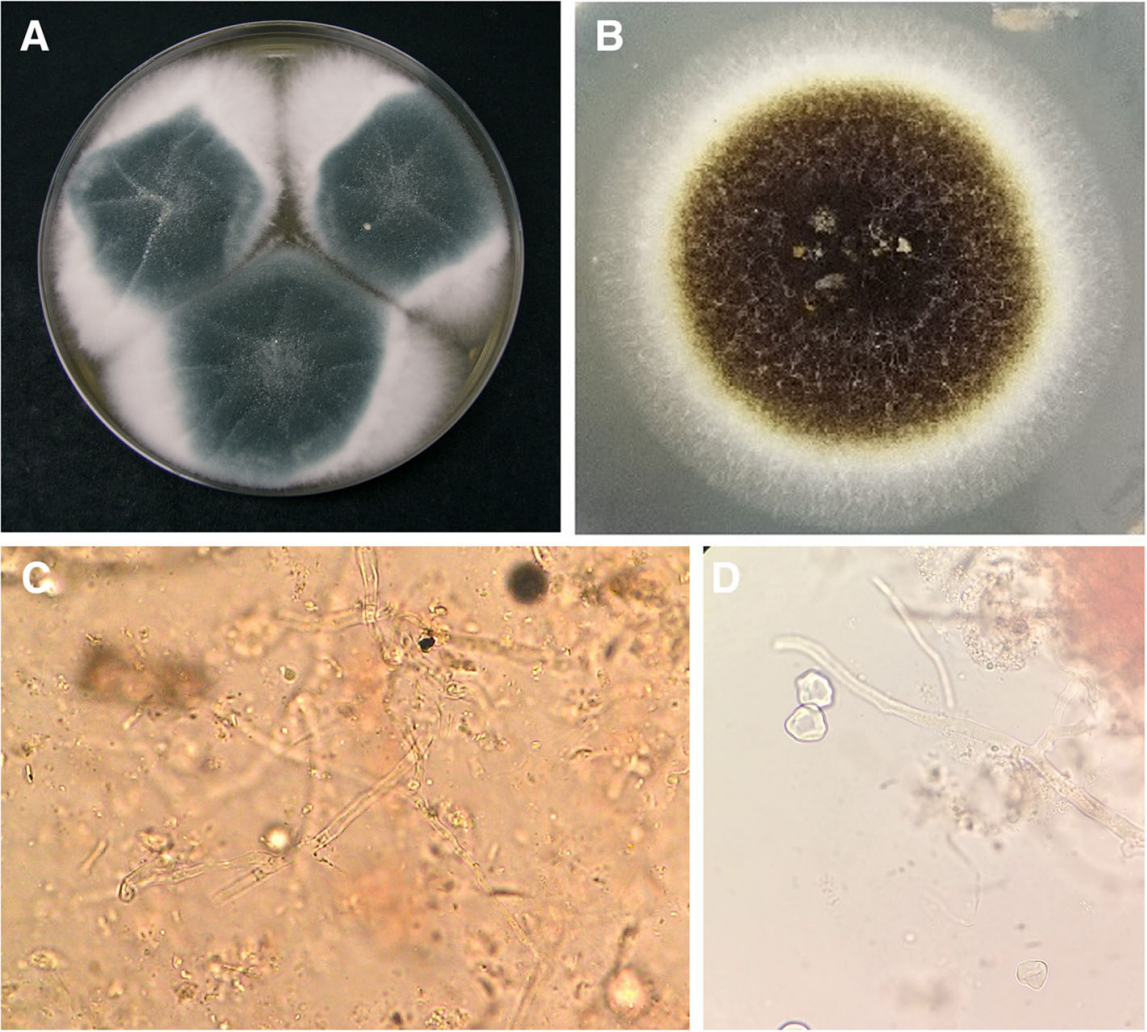
Isolated *Aspergillosis fumigatus* in BAL sample (**a**) and *Aspergillus niger* in the brain tissue biopsy (**b**), direct microscopic examination in KOH preparation in brain biopsy X40 (**c** and **d**)

Intravenous voriconazole started and diagnostic bronchoscopy and bronchoalveolar Lavage (BAL) scheduled. The result of BAL culture was positive for *A. fumigatus* on sabouraud dextrose agar (Merck, Germany) (Fig. 4). The minimum inhibition concentrations (MIC) for amphotericin B, caspofungin, voriconazole, posaconazole, and itraconazole for *A. fumigatus* were 4 mg/l, 0.032 mg/l, 0.25 mg/l, 0.032 mg/l, and 0.125 mg/l, respectively.

**Fig. 5:**
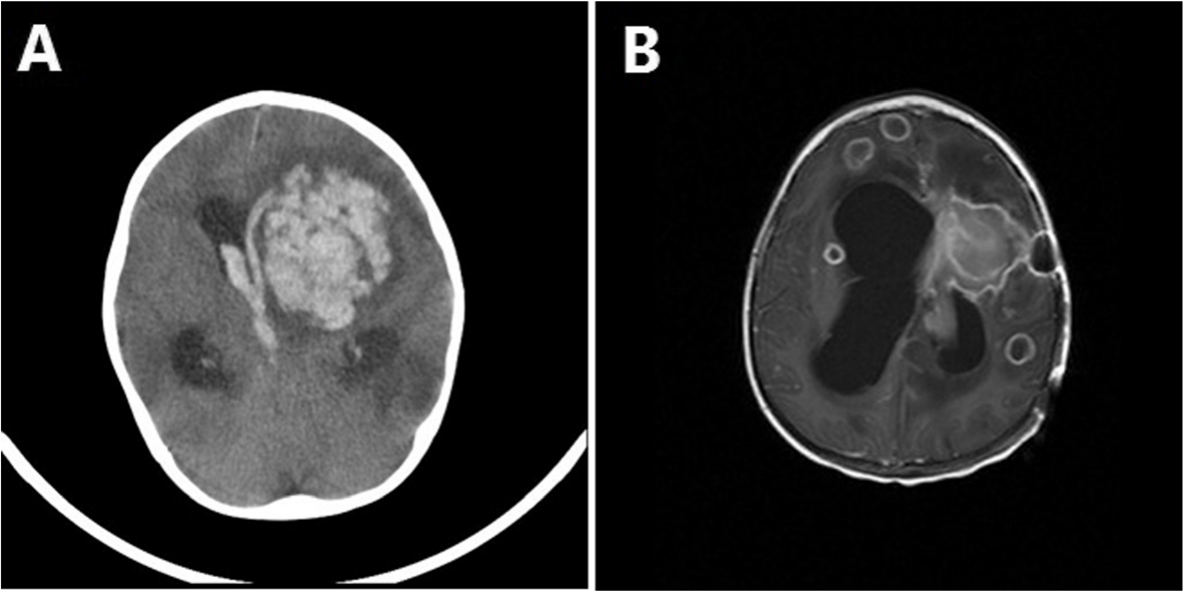
Extensive left hemisphere intracerebral hemorrhage in the first brain CT scan (**a**) and multiple varying size ring like enhancing lesions within the different parts of white matter of both cerebral and cerebellar hemispheres representing abscess formation (**b**)

A few days later, the patient’s condition deteriorated suddenly by abnormal focal movement in his right upper limb, loss of consciousness (Glasgow Coma Scale: 4), and seizure. He transferred to the pediatric intensive care unit. The cerebrospinal fluid (CSF) analysis revealed total cell count 15 without WBC, protein 164 mg/dl, Lactate dehydrogenase 65 U/L (normal range < 70 U/L), and sugar 48 mg/dl. Brain CT scan showed extensive left hemisphere intracerebral hemorrhage (Fig. 5a). A pediatric neurosurgeon provided external drainage and hemorrhage drained. Fungal PCR and culture from CSF requested, all with negative results. Caspofungin (Cancidas) was added to his antifungal regimen because mucormycosis or fusariosis rarely develop during Ambisome prophylaxis [11]. Brain magnetic resonance imaging (MRI) revealed multiple brain abscesses (Fig. 5b).

Brain biopsy performed because of persistent fever despite extensive medical and surgical treatment. KOH revealed septate hyphae (Fig. 4). Tissue biopsy cultured, which was positive for mold infection, the isolated species identified by beta-tubulin gene and sequencing [12]. All other requested diagnostic tests and bacterial culture on brain biopsy were negative. The beta-tubulin gene sequence result was compared with the GenBank database (www.ncbi.nlm.nih.gov) and revealed that the isolated species is *Aspergillus niger*. It deposited in the GenBank database with accession number MT561886. The MIC for amphotericin B, caspofungin, voriconazole, posaconazole, and itraconazole in isolated *A. niger* were 8 mg/l, 0.032 mg/l, 0.5 mg/l, 0.032 mg/l, and 0. 25 mg/l. The patient stayed in the hospital for several weeks without any changes in his clinical status and died due to sudden cardiac arrest associated with severe CNS *Aspergillus* infection, renal impairment, and septic shock 142 days after admission and 108 days after AF therapy.

## Discussion and conclusions

Patients with hematological malignancies are at increased risk of various infections, including IFIs. The risk is higher among those receiving chemotherapy for acute myeloid leukemia, myelodysplastic syndrome, ALL, patients during induction-remission chemotherapy, graft versus host disease, and corticosteroid therapy. AF prophylaxis recommended in high-risk patients in the presence of prolonged severe neutropenia (ANC < 500 cells/μL > 7 days) [13]. Even with the first-line choices, AF prophylaxis may be unsuccessful in about 3–14% of the patients at risk of IFIs [14–16]. The occurrence of bIFIs during primary AF prophylaxis at initial remission-induction chemotherapy is considered as “early bIFIs”, while “late bIFIs” usually develops during re-induction for relapsed leukemia, and prolonged corticosteroid treatment [15].

There are some risk factors for developing bIFIs like the occurrence of IFIs due to resistant pathogens, development of resistance in previously susceptible fungi, inadequate absorption (mucositis, enteritis), abnormal metabolism (CYP2C19 heterogenicity), ineffective distribution, drug interactions, and conditions that perpetuate the infection (refractory/relapsed hematological illness). Although *Mucormycosis* is considered a common cause of bIFIs in those on azole prophylaxis in some reports, other mold infections such as *Aspergillus* and non-*Aspergillus* species are also well-known as etiologic agents [14]. The proportion of bIFIs caused by mold varies between 27 and 73% [17–24]. The prevalence of bIFIs may also be affected by the AF class. For example, bIFIs in the patients treated with caspofungin usually are caused by *Aspergillus* spp*.* [25]. Data regarding post-amphotericin prophylaxis bIFIs are limited, but *Aspergillus* spp*.* (*A. terreus*), *Fusarium* spp., *Scedosporium* spp. and even *Mucorales* have been reported [23].

The emergence of azole-resistant pathogens warrants careful adherence to the stewardship programs in high-risk settings such as hematology/oncology wards [26, 27]. The reported prevalence of azole-resistant strains is more than 50% in the literature [14]. However, the rate is much lower for other AF agents such as amphotericin. In the present case, pulmonary IA developed 24 days after AF prophylaxis, during induction-remission chemotherapy.

Early diagnosis and antifungal therapy are vitally crucial for the best outcome of the patient. The treatment of infections caused by fungi may differ, so it is essential to confirm the genus by culture, PCR, and sequencing. Both microscopic examination and culture are insensitive, and therapy should not withhold in the absence of such confirmation. Non-invasive methods like serum biomarkers (GM and beta-D-glucan assays) are established for the diagnosis of IA [28]. Molecular techniques can also help the clinicians to detect fungal infections in the early stages of IA [29, 30]. The monitoring of both serum GM and blood-PCR is associated with an earlier diagnosis of IA [31]. There are limited reports regarding the diagnosis of CNS infections using the GM assay and PCR. In our case, the GM test was positive in both CSF and blood, while real-time PCR was positive only in the former. Nevertheless, both tests were positive in the third CSF sample, indicating that an accurate diagnosis demands multiple sampling.

In this study, two *Aspergillus* species isolated from the patient. Diagnosis of *A. niger* is difficult because *Aspergillus* section *Nigri* is a pigmented fungus (also called black *Aspergilli*) and morphologically is very similar to *A. niger*. Simões and coworkers by colony morphologic characterizing, microscopic examination, and spectral mass analyses were reported species of *Aspergillus* section *Nigri* including *Aspergillus aculeatus, Aspergillus brasiliensis, Aspergillus carbonarius, Aspergillus ellipticus, Aspergillus ibericus, Aspergillus japonicas, Aspergillus lacticoffeatus, Aspergillus niger, Aspergillus phoenicis, Aspergillus sclerotioniger, Aspergillus tubingensis, Aspergillus uvarum,* and *Aspergillus vadensis* [32]. The infections caused by *Aspergillus* section *Nigri* are rare and more of these were categorized in *A. niger* infections. Gautier and coworker reported, from 85 *A. niger* isolated from respiratory samples, 40 species were diagnosed as *A. tubingensis* by matrix-assisted laser desorption/ionization time-of-flight mass spectrometry, infected patients suffered from different types of chronic respiratory failure [32]. Therefore, *Aspergillus niger* identified by beta-tubulin gene and sequencing in this case.

In our case, although both isolates showed low MIC to all tested Azoles, high MIC to amphotericin B was found. Amphotericin resistant *A. niger* documented in the present case report warrants special consideration in high-risk patients receiving amphotericin B for prophylaxis. Thus, given the different AF susceptibility patterns, identification of the *Aspergillus* at the species level is suggested [13, 33] and should be considered in multiple site involvement.

CNS infections may happen as an occult asymptomatic extra-pulmonary involvement during the diagnostic evaluation of febrile neutropenic patients or symptomatic form, which usually developed after a few weeks (median of 2 weeks) of pulmonary manifestation (range: 5–283 days) [34]. In our case, the patient’s first neurological signs and symptoms were developed as the consequence of extensive CNS hemorrhage concomitantly with pulmonary involvement (three days later). Serial neuroimaging studies revealed secondary fungal abscess formation at the site of hemorrhagic infarctions, which is very rare but previously described [35]. Clinical presentations of fungal brain abscess may not be specific and primarily depends on the site of involvement, single versus multiple lesions and pathological processes (angioinvasion, thrombosis or hemorrhage) [19]. The most common manifestations of fungal CNS infections include fever, nausea, vomiting, altered mental status (confusion, lethargy or loss of consciousness), seizure, focal neurological deficits, tremor, and ataxia [24]. Symptoms may progress rapidly in severely immunocompromised hosts.

Data regarding mixed mold infections are somewhat lack in the literature. Based on the most recent report by Magira et al., *Aspergillus* spp. was the most prevalent type of identified mixed mold infection in 27 patients retrospectively studied. The most common reported combinations were *A. fumigatus*/*A. terreus,* and *A. terreus*/*A. niger*, with the mortality rate in such infections being as high as 70% [36]. Mixed mold infection is an infrequent phenomenon, representing a diagnostic and treatment challenge, especially in immunocompromised hosts. There are limited data in the literature about the combination of *A. fumigatus*/ *A. niger* in children with vital organ involvement. The definitive diagnosis of CNS aspergillosis requires a high index of suspicion and an aggressive approach. We present herein a case of mixed *A. fumigatus*/ *A. niger* infection with fatal outcome in a patient with ALL. Given the different AF susceptibility patterns, identification of the *Aspergillus* at the species level should considered in invasive fungal infections with multiple site involvement. In line with the current ESCMID-ECMM-ERS guideline, consideration of different AF classes for the treatment of bIFIs in the patients receiving amphotericin B warranted.

## Data Availability

All data generated or analyzed during this study are included in this published article.

## Abbreviations

CNS: Central nervous system
AF: Antifungal
ALL: Acute lymphoblastic leukemia
bIFI: Breakthrough invasive fungal infection
WBC: White blood cell count
GM: Galactomannan
PCR: Polymerase chain reaction
CT: Computed tomography
CSF: Cerebrospinal fluid
MRI: Magnetic resonance imaging
ANC: Absolute neutrophil count
HIV: Human immunodeficiency virus infections
IA: Invasive aspergillosis
AMOC: Amir medical oncology center
